# Foundation Meeting

**Published:** 1919-07-26

**Authors:** 


### THE AMERICAN HOSPITAL FOR GREAT BRITAIN.

### Foundation Meeting.

WHAT will prove to be a notable addition to the hospitals in this country—probably in London—was formally initiated at an influential meeting held in the Council-chamber of the Royal Society of Medicine on Thursday, July 17, when the American Hospital for Great Britain was legally launched.

**The Right Hon. the Earl of Reading, P.C.** (Lord Chief Justice of England), presided. He said that the co-operation which had existed between Americans and Britons during the last two years of the war had been notably demonstrated in the relations between the medical and surgical profession in the two countries; its members on both sides of the Atlantic, by being brought into close contact with each other during that time, had learned to appreciate more widely the methods of each. And now the generous and benevolent idea on the part of medical men and distinguished citizens in America, of establishing an American hospital in this country in order to mark the new era of a better understanding, was to receive sanction and support at this meeting. He was glad to say that Britain had not fallen short in the matter of extending hospitality to Americans in this country, particularly if they fell ill during their visit, and it was not really necessary for America to mark her stand in this country by providing her own hospital. At the same time, Britons would recognise the gracious idea which had occurred to the gentlemen who were responsible for the project, and he thought the prime motive was a better knowledge and closer contact between the members of the profession of the two countries and the fostering of mutual goodwill. Previously, it had been the custom of Americans desiring to take post-graduate courses to do so in Berlin, Vienna, or other foreign city, but the present movement would enhance the excellent opportunities afforded by London for these studies, and familiarity with the language would be an added advantage, in addition to which a large number of the population would be found ready to extend to them the most generous hospitality in their power. The hospital would promote scientific study and research, and would be open to patients of all classes, irrespective of creed or nationality. He believed the new hospital would have a really marked effect on the future relations between the two countries, and that those who were now responsible for its inception would, in the time to come, congratulate themselves on having taken a part in it.

**Mr. Philip Franklin, F.R.C.S.**, read to the meeting a report in which he related the preliminary steps that had been taken towards the establishment of the hospital. There was full appreciation of the fact that a number of beds were allocated to Americans in London in various hospitals, but it was felt that this was not enough. There was no hospital conducted on American lines in this capital where the citizen from the States could feel he was being cared for after the manner obtaining in his own land: the little details which gave a home feeling, and the importance of which was apt to be exaggerated by those who were reduced by sickness. This the new hospital would provide. In the past Americans who wished to come to Europe for specialised and advanced medical study had not selected London to any extent, yet the wealth of clinical material available in this city was unequalled by that of any other centre in Europe. This was not just his own view, but was the unanimous verdict of two hundred American medical officers who had been working here. The American medical man was likely to visit London more in the future than he had done in the past, and the new hospital would form his appropriate medical home.

The report was adopted.

**Mr. R. Newton Crane** (in the absence of the American Ambassador) moved:

"That, in commemoration of the co-operation of the medical men of the United States and Great Britain during the European War, and to strengthen the friendship existing between the two notions, the American Hospital for Great Britain be, and is hereby, founded for the medical and surgical treatment of patients of all classes, irrespective of creed or nationality, and for the promotion of scientific study and research."

He said the hospital would be a powerful factor in drawing together the peoples of the United States and this country. Much of the pro-German propaganda by native Americans during the war was traceable to the friendships formed between American medical men and Germans and Austrians whilst the former were walking the continental hospitals. That led to the belief that those were the only countries where anything like warm friendship for America could be found. As an illustration of that feeling he mentioned the fact that, a few months ago, a distinguished ambassador returned to this country from his post in Russia, and when he arrived here it was found necessary that he should undergo an operation. The speaker was consulted by him as to how he could get back to America or to the Continent. When told that there were physicians and surgeons in London the equal of any in the world he was incredulous, and went to France to meet a surgeon from Johns Hopkins University, who operated upon him.

**Sir W. Arbuthnot Lane,** in seconding the resolution, said that during his recent visit to the Congress in Atlantic City he took every opportunity of conversing with important American surgeons and physicians, and found them all delighted to receive this new hospital, as they realised it would not only furnish a means for the education of Americans here, but would bring together the two races in a manner that no other means would accomplish.

The resolution was carried.

**Sir Humphry Rolleston, K.C.B.** (President of the Royal Society of Medicine), proposed:

"That a Governing Council be constituted, and that the following gentlemen be the original members of the Council: His Excellency the American Ambassador, Walter Blackman, Esq., George M. Cassatt, Esq., R. Newton Crane, Esq., Wilson Cross, Esq., James E. Dunning, Esq., J. Grant Forbes, Esq., Philip Franklin, Esq., Clarence Graff, Esq., Robert Grant, Esq., jun., the Consul-General, James Benson Kennedy, Esq., J. Blair MacAfee, Esq., George A. Mower, Esq., Francis E. Powell, Esq., H. Gordon Selfridge, Esq., Henry E. Stoner, Esq., Lawrence L. Tweedy, Esq., F. C. Van Duzer, Esq., E. Bradner White, Esq., the Hon. Robt. E. Skinner."

He said that now the decision to found a hospital had been taken it was important that steps should at once be proceeded with to make this institution worthy of the great nation after which it had been named. Much labour and organisation were required, and medical men as a rule were so engrossed in their profession that they seldom had time to devote to large movements. He hoped the gentlemen whose names were included in the resolution would consent to give their valuable services and form the original members of the Governing Body.

**Sir John Bland-Sutton** seconded the resolution. He said the first essential in such an undertaking was, not to have a good medical and surgical staff, but a good lay board. Moreover, doctors were usually bad organisers, though there were outstanding instances to the contrary. With the exception of vaccination, he thought the discovery of coal-tar products and the invention of the compound microscope had done more to advance the science of medicine and surgery than had anything else. In the foundation of this new hospital there was afforded an opportunity of having a building on modern lines which would be good for the sick and suffering, good for the education of medical men, and for discovering the causes and the means of prevention of disease. He hoped it would furnish as fine a

**The American Hospital for Great Britain**—(*cont.*).

school as any in London, as fine a place for research as any in the world. It was impossible to divorce the discovery of the cause of disease from places where the sick congregated. All the hospitals in London had undergone an extraordinary modification as the result of modern progress in medicine and surgery, and there was need to keep pace with the great advances in thought and discovery.

The resolution was carried.

**Mr. Francis E. Powell** moved:

"That Medical Committees in Great Britain and the United States be constituted, and that the following gentlemen be the original members of the said Committees:

"Of the Medical Committee in Great Britain: Sir William Osler, Bt., M.D., F.R.S., F.R.C.P., Regius Professor of Medicine, University of Oxford; Sir Arbuthnot Lane, Bt., C.B., M.B., M.S., F.R.C.S.; Sir Humphry Rolleston, K.C.B., M.A., M.D., F.R.C.P., President of the Royal Society of Medicine; Sir John Bland-Sutton, F.R.C.S., Vice-President of the Royal College of Surgeons; J. Y. W. MacAlister, Esq., F.S.A., Secretary of the Royal Society of Medicine; Philip Franklin, Esq., F.R.C.S., Joint Honorary Secretary of the Fellowship of Medicine; with power to add to their number.

"Of the Medical Committee in the United States: Dr. George W. Crile, of Cleveland, nominated by the American Academy of Science on International Relations; Dr. W. J. Mayo, of Rochester, Minnesota; Dr. Charles H. Mayo, of Rochester, Minnesota; Dr. Albert J. Ochsner, of Chicago; Dr. Rudolph Matas, of New Orleans; Dr. Franklin Martin, of Chicago, nominated by the American Gynecological Association; with power to add to their number."

He said that in such an institution as this it was of the utmost necessity to have a staff attached to the hospital which would carry conviction to the respective students and physicians from America who wished to take the courses; and the list he submitted provided that assurance.

**Mr. Walter Blackman** seconded, and it was carried.

**Sir W. Arbuthnot Lane** proposed, and **Mr. F. C. Van Duzer** seconded, the appointment of Mr. Philip Franklin as Honorary Secretary of the hospital. Sir Arbuthnot said he had been associated, in a secondary manner, with Mr. Franklin in the initiation of this hospital, and had been much struck with his energy, pertinacity, and determination.

**Mr. Wilson Cross** moved:

"That a Committee be appointed to draft the laws and constitution of the hospital, and to report to an early meeting of the Council as to the advisability of incorporation, and the appointment of treasurer, banker, solicitor, and secretary."

He considered it, he said, a wonderful privilege for a business man to ally himself with a movement of this kind. The names which had been suggested were as follows: Mr. R. Newton Crane, Mr. E. Bradner White, Mr. Forbes, Mr. Van Duzer, Mr. Geo. A. Mower, and Mr. Geo. M. Cassatt, with power to add to their number.

**Mr. J. Grant Forbes** seconded, and it was carried.

**The Chairman** suggested that a number of distinguished men in England should be invited to become Vice-Presidents of the Society. It would be undesirable to mention names until the gentlemen concerned had been approached and their consent obtained, but he suggested the appointment of a small Committee for this purpose. It was agreed to leave this matter in the hands of the Chairman, Mr. Newton Crane, and Mr. Franklin.

**Mr. Geo. M. Cassatt** moved, and **Mr. Geo. A. Mower** seconded, a proposal to form a Finance and Appeal Committee, consisting of Mr. F. E. Powell, Mr. Robt. Grant, Mr. J. E. Dunning, Mr. J. B. Kennedy, and Mr. Wilson Cross, with power to add to their number. This was agreed to, as was also a resolution of thanks to the Council of the Royal Society of Medicine for the use of the meeting-room.

**Sir Humphry Rolleston**, in acknowledging the last-named vote, desired to remove a misapprehension which, he thought, existed in some quarters to the effect that the projected hospital would interfere with the arrangements for teaching which had been made by the Post-Graduate Association. The exact reverse of this was the truth; the new hospital would constitute a centre from which American medical men in this country could take part in the activities of the Association. He referred to the subject, as he feared it had been a ground of misconception.

A resolution thanking Lord Reading for presiding over this meeting, and for the energy he had displayed in the formation of the new hospital, was heartily carried.

**Lord Reading**, in his brief reply, said he hoped this meeting would prove to be the forerunner of many useful ones in the future.

